# Frequency-Locked Detector Threshold Setting Criteria Based on Mean-Time-To-Lose-Lock (MTLL) for GPS Receivers

**DOI:** 10.3390/s17122808

**Published:** 2017-12-04

**Authors:** Tian Jin, Heliang Yuan, Na Zhao, Honglei Qin, Kewen Sun, Yuanfa Ji

**Affiliations:** 1 School of Electronic and Information Engineering, Beihang University, Beijing 100191, China; jintian@buaa.edu.cn (T.J.); yuanheliang@buaa.edu.cn (H.Y.); zhaona@buaa.edu.cn (N.Z.); 2 School of Computer and Information, Hefei University of Technology, Hefei 230009, China; kewen.sun@hfut.edu.cn; 3 Guangxi Key Laboratory of Precision Navigation Technology and Application, Guilin 541004, China; jiyuanfa@163.com

**Keywords:** GPS receiver, frequency locked loop, frequency-locked detector, mean-time-to-lose-lock

## Abstract

Frequency-locked detector (FLD) has been widely utilized in tracking loops of Global Positioning System (GPS) receivers to indicate their locking status. The relation between FLD and lock status has been seldom discussed. The traditional PLL experience is not suitable for FLL. In this paper, the threshold setting criteria for frequency-locked detector in the GPS receiver has been proposed by analyzing statistical characteristic of FLD output. The approximate probability distribution of frequency-locked detector is theoretically derived by using a statistical approach, which reveals the relationship between probabilities of frequency-locked detector and the carrier-to-noise ratio (*C*/*N*_0_) of the received GPS signal. The relationship among mean-time-to-lose-lock (MTLL), detection threshold and lock probability related to *C*/*N*_0_ can be further discovered by utilizing this probability. Therefore, a theoretical basis for threshold setting criteria in frequency locked loops for GPS receivers is provided based on mean-time-to-lose-lock analysis.

## 1. Introduction

In the Global Positioning System (GPS) receiver, the lock detector is usually used to indicate the signal tracking status by comparing with a threshold. Code, frequency and phase are tracked by delay, frequency, and phase locked loops, respectively. These loops have their own indicators [1,2]. The GPS receiver mainly relies on phase locked loop (PLL) and delay locked loop (DLL) to track signal and frequency locked loop (FLL) is a transition part to bridge acquisition and PLL + DLL. However, in high performance GPS receivers, such as high dynamic and high sensitivity receivers, the FLL has been widely used when the carrier phase could not be tracked by PLL or aids PLL to track signal [3,4]. It indicates the significance of analyzing frequency-locked detector. For example, a detailed discussion and comparison of PLL and FLL can be found in [5]. Yang and Huang [6] proposed a non-linear carrier NCO unit to track carrier precisely by selecting interpolating filter orders, which is derived from the frequency discriminator in high dynamic situation. Curran et al. [7] discussed the design and steady-state performance of one first-order, two second-order FLL loop filters, and four carrier frequency discriminators, then a new FLL design under weak signal conditions was proposed. Meanwhile, FLLs have some distinct advantages over PLL counterparts [8]. By neglecting absolute phase error and permitting relative phase rotation of the received signal and the local carrier replica, an FLL can, typically, acquire and track signals that are at higher frequency offsets than a PLL. It is easy to understand that FLL plays a key role in facilitating reliable signal tracking. The frequency-locked detector (FLD) is used to indicate FLL signal tracking state. Natali [9] presented several AFC loops and performance for different signal. Messerschmi [10] has described two simply implemented frequency detectors to aid PLL timing and carrier acquisition, even with very small loop bandwidths and large initial frequency offsets. Mileant and Hinedi [11] proposed an FLD based on square law, which experiences less degradation due to phase jitter than the absolute value detector. Linn and Peleg [12] suggested a family of PLDs for M-PSK receivers operating in additive white Gaussian noise channels and derived the statistical properties of PLD. Kratyuk et al. [13] discussed Frequency detector for fast frequency lock of digital PLLs, which provides frequency difference information at each reference cycle to guarantee fast frequency acquisition. Previous researches mainly focus on the structure of FLL and its detector, but how to set the FLL detector threshold is seldom discussed, which make it hard to indicate the tracking frequency is stabilized or not. Our main purpose is to analyze the statistical performance of FLD by mean-time-to-lose-lock approaches, and make comparison with PLD, which has been studied by Jin et al. [14].

## 2. Frequency-Locked Detector Output $C_{2\varphi }$

In the GPS receiver, the GPS signal received by the antenna is down converted to intermediate frequency through low noise amplifier, filters, and down-converter [15], the obtained signal S(t) can be written as follows:

(1) $$S(t)=AC(t)D(t)\cos[(\omega_{IF}+\omega_{Doppler})t+\phi_{0}]+n(t)$$

where A is the signal amplitude, C(t) is PRN code of GPS, D(t) is the navigation data, $\omega_{IF}$ is the intermediate frequency, $\omega_{Doppler}$ is the Doppler frequency shift, $\phi_{0}$ is the initial carrier phase, n(t) is the additive white Gaussian noise.

According to [4,16,17], LOS (line of sight) signal through the front-end *A*/*D* sampling, quantization and integration, the in-phase, and quadrature-phase integrations at ith interval are *I_i_* and *Q_i_*, which are shown as:

(2) $$\begin{array}{cc} I_{i} & \approx \sqrt{2\frac{C}{N_{0}}T_{coh}}R(\tau )\sin c(\pi \Delta f_{i}T_{coh})\cos(\pi \Delta f_{i}T_{coh}+\Delta \phi_{i})+n_{I,i}\\ & =\sqrt{2\frac{C}{N_{0}}T_{coh}}R(\tau )\sin c(\pi \Delta f_{i}T_{coh})\cos(\Delta \theta_{i})+n_{I,i}\\ & =A_{i}\cos(\varphi_{i}) \end{array}$$

(3) $$\begin{array}{cc} Q_{i} & \approx \sqrt{2\frac{C}{N_{0}}T_{coh}}R(\tau )\sin c(\pi \Delta f_{i}T_{coh})\sin(\pi \Delta f_{i}T_{coh}+\Delta \phi_{i})+n_{Q,i}\\ & =\sqrt{2\frac{C}{N_{0}}T_{coh}}R(\tau )\sin c(\pi \Delta f_{i}T_{coh})\sin(\Delta \theta_{i})+n_{I,i}\\ & =A_{i}\sin(\varphi_{i}) \end{array}$$

where $C/N_{0}$ is carrier to noise ratio, $T_{coh}$ is coherent integration time, R(τ) is the auto-correlation function of PRN code, τ is the code phase delay between received and local replica, $\Delta f_{i}$ and $\Delta \phi_{i}$ are the frequency error between received and local replica and the initial phase error between received and local replica, $\Delta \theta_{i}$ is total phase error that includes $\Delta f_{i}$ and $\Delta \phi_{i}$ in the *i*th intervals. $n_{I,i},n_{Q,i}$ are independent white Gaussian noises corresponding to the *I* and *Q* branches, respectively. For the convenience of calculation, we take normalization processing of $n_{I,i}\text{\textasciitilde{}}N(0,1),n_{Q,i}\text{\textasciitilde{}}N(0,1)$ [14]. $A_{i}$ and $\varphi_{i}$ are signal amplitude and average phase estimation, containing noise component, relative to $\sqrt{2\frac{C}{N_{0}}T_{coh}}R(\tau )\sin c(\pi \Delta f_{i}T_{coh})$ and $\Delta \theta_{i}$. Detailed discussions of the effects of signal quantization are given in [18,19].

The output of frequency-locked detector during a period of time was proposed by [20], which is shown as:

(4) $$C_{2\varphi }=\frac{1}{M}\sum_{i=1}^{M}\frac{D_{i}}{P_{i}}$$

where $D_{i}={\left(Dot\right)}^{2}-{\left(Cross\right)}^{2}$, $P_{i}={\left(Cross\right)}^{2}+{\left(Dot\right)}^{2}$, $Dot+jCross=(I_{i}+jQ_{i})(\bar{I_{i-1}+jQ_{i-1}})$. *M* is the times of accumulation.

Further, according to Spiegel [21], (4) can be simplified as:

(5) $$D_{i}={\left(Dot\right)}^{2}-{\left(Cross\right)}^{2}=[{\left(Dot\right)}^{2}+{\left(Cross\right)}^{2}]\cos(2\varphi_{i}-2\varphi_{i-1})$$

(6) $$\begin{array}{cc} C_{2\varphi } & =\frac{1}{M}\sum_{i=1}^{M}\frac{D_{i}}{P_{i}}=\frac{1}{M}\sum_{i=1}^{M}\frac{{\left(D{ot}_{i}\right)}^{2}-{\left(Cross_{i}\right)}^{2}}{{\left(Dot_{i}\right)}^{2}+{\left(Cross_{i}\right)}^{2}}\\ & =\frac{1}{M}\sum_{i=1}^{M}\frac{\left[{\left(Dot_{i}\right)}^{2}+{\left(Cross_{i}\right)}^{2}]\cos(2\varphi_{i}-2\varphi_{i-1}\right)}{{\left(Dot_{i}\right)}^{2}+{\left(Cross_{i}\right)}^{2}}\\ & =\frac{1}{M}\sum_{i=1}^{M}\cos(2\varphi_{i}-2\varphi_{i-1})\\ & =\frac{1}{M}\sum_{i=1}^{M}\cos(2\Delta \varphi_{i}) \end{array}$$

where $\varphi_{i-1}$ and $\varphi_{i}$ are average phase estimation errors in the (*i* − 1)th and *i*th intervals, respectively. $\Delta \varphi_{i}$ is the difference between $\varphi_{i}$ and $\varphi_{i-1}$.

## 3. Distribution of FLD Output $C_{2\varphi }$

According to Zhuang [22], the statistical properties of φ obeys Rician distribution with |φ|≤π, its probability density function is

(7) $$f(\varphi )=\frac{1}{2\pi }e^{-\frac{C}{N_{0}}T_{coh}}\left[1+\sqrt{2\frac{C}{N_{0}}T_{coh}}\cos(\varphi -\Delta \theta )\exp(\frac{C}{N_{0}}T_{coh}\cos^{2}(\varphi -\Delta \theta ))\int_{-\infty }^{a}e^{-\frac{x^{2}}{2}}dx\right]$$

where $a=\cos(\varphi -\Delta \theta )\sqrt{2\frac{C}{N_{0}}T_{coh}}$. The approximate simplification of (7) shows that φ is nearly subjected to Gaussian distribution under high $C/N_{0}$, while it obeys uniform distribution under low $C/N_{0}$. Based on the different distribution of φ, under different $C/N_{0}$ conditions, we can analyze the distribution characteristics of FLD output $C_{2\varphi }$ individually.

### 3.1. $C_{2\varphi }$ Distribution under Frequency Lock

When frequency locked loop is in lock and $C/N_{0}$ is high, we can make the following assumptions:

(8) $$\exp(-\frac{C}{N_{0}}T_{coh})\approx 0$$

(9) $$\cos^{2}(\varphi -\Delta \theta )=1-\sin^{2}(\varphi -\Delta \theta )\approx 1-{\left(\varphi -\Delta \theta \right)}^{2}$$

(10) $$\int_{-\infty }^{\cos(\varphi -\Delta \theta )\sqrt{2\frac{C}{N_{0}}T_{coh}}}e^{-\frac{x^{2}}{2}}dx\approx \sqrt{2\pi }$$

Based on the previous assumptions, (7) can be simplified to

(11) $$f(\varphi )\approx \frac{1}{\sqrt{2\pi *(\frac{1}{2T_{coh}C/N_{0}})}}*\exp(-\frac{{\left(\varphi -\Delta \theta \right)}^{2}}{2*(\frac{1}{2T_{coh}C/N_{0}})})|\varphi |\leq \pi$$

where φ follows Gaussian distribution with the mathematical expectation Δθ and variance $1/(2T_{coh}\cdot \frac{C}{N_{0}})$ ($\sigma^{2}=1/(T_{coh}\cdot \frac{C}{N_{0}})$). Then, we can get the probability distribution function of Δφ shown below. When considering noise of *i*th and (*i* − 1)th are independent with each other, $\varphi_{i}$ and $\varphi_{i-1}$ are independent of each other, while $\Delta \theta_{i}$ and $\Delta \theta_{i-1}$ (mathematical expectation of $\varphi_{i}$ and $\varphi_{i-1}$) are approximately equal to zero under frequency lock and interval is small. Thus, we can calculate the mathematical expectation $E\{\Delta \varphi_{i}\}$ and variance $VAR\{\Delta \varphi_{i}\}$, and probability distribution function of Δφ, which are shown as follows:

(12) $$E\{\Delta \varphi_{i}\}=E\{\varphi_{i}\}-E\{\varphi_{i-1}\}=\Delta \theta_{i}-\Delta \theta_{i-1}\approx 0$$

(13) $$VAR\{\Delta \varphi_{i}\}=VAR\{\varphi_{i}-\varphi_{i-1}\}=VAR\{\varphi_{i}\}+VAR\{\varphi_{i-1}\}=\sigma^{2}$$

(14) $$f(\Delta \varphi_{i})=\int_{-\infty }^{+\infty }f(\Delta \varphi_{i}-\varphi_{i-1},\varphi_{i-1})d\varphi_{i-1}=\frac{1}{\sqrt{2\pi }\sigma }\exp(-\frac{\Delta \varphi_{i}^{2}}{2\sigma^{2}})$$

The distribution characterization of FLD output represents a Gaussian stochastic process through a cosine system. We can get the mathematical expectation and variance of $C_{2\varphi }$ in (6) as follows:

(15) $$\begin{array}{cc} E\{C_{2\varphi }\} & =E\{\frac{1}{M}\sum_{i=1}^{M}\cos(2\Delta \varphi_{i})\}\\ & =\frac{1}{M}\sum_{i=1}^{M}\int_{-\infty }^{\infty }\cos(2\Delta \varphi_{i})p(\Delta \varphi_{i})d\Delta \varphi_{i}\\ & \approx \frac{1}{\sqrt{2\pi }\sigma }\int_{-\infty }^{\infty }\cos(2\Delta \varphi_{i})\exp(-\frac{\Delta \varphi_{i}^{2}}{2\sigma^{2}})d\Delta \varphi_{i}\\ & =\exp(-2\sigma^{2}) \end{array}$$

(16) $$\begin{array}{cc} VAR\{C_{2\varphi }\} & =VAR\{\frac{1}{M}\sum_{i=1}^{M}\cos(2\Delta \varphi_{i})\}\\ & =\frac{1}{M^{2}}\sum_{i=1}^{M}VAR\{\cos(2\Delta \varphi_{i})\}\\ & =\frac{E\{\cos^{2}(2\Delta \varphi )\}-E^{2}\{\cos(2\Delta \varphi )\}}{M}\\ & =\frac{1}{M}\frac{1}{\sqrt{2\pi }\sigma }\int_{-\infty }^{\infty }\cos^{2}(2\Delta \varphi )\exp(-\frac{\Delta \varphi^{2}}{2\sigma^{2}})d\Delta \varphi -\frac{1}{M}\exp(-4\sigma^{2})\\ & =\frac{1}{2M}(1+e^{-8\sigma^{2}})-\frac{1}{M}e^{-4\sigma^{2}} \end{array}$$

When the signal is locked, $\Delta \varphi_{i}$ is stable around zero. Therefore, $C_{2\varphi }$ approximates to

(17) $$C_{2\varphi }=\frac{1}{M}\sum_{i=1}^{M}\cos(2\Delta \varphi_{i})\approx 1-\frac{2}{M}\sum_{i=1}^{M}\Delta \varphi_{i}^{2}$$

For simplification, we can assume a new variable *Y* related with $C_{2\varphi }$, which nearly follows the chi-square distribution

(18) $$Y=\frac{(1-C_{2\varphi })M}{2\sigma^{2}}=\sum_{i=1}^{M}{\left(\frac{\Delta \varphi_{i}}{\sigma }\right)}^{2}\sim \chi^{2}(M)$$

According to (18), the probability distribution function of FLD output is shown as:

(19) $$f_{C_{2\varphi }}(c_{2\varphi })=\frac{M}{2\sigma^{2}}f_{Y}\left(\frac{M-Mc_{2\varphi }}{2\sigma^{2}}\right)\;|c_{2\varphi }|\leq 1$$

where $f_{Y}(y)$ is the probability distribution function of the random variable Y($Y\sim \chi^{2}(M)$).

### 3.2. $C_{2\varphi }$ Distribution under Frequency Unlock

Under low C/N_0_ condition, frequency loop may be unlocked. After simplification of Equation (7), φ is uniformly distributed in (−π,π), and its probability distribution function is shown as:

(20) $$f(\varphi )\approx \frac{1}{2\pi }\;|\varphi |\leq \pi$$

When *M* = 1, the probability distribution functions of Δφ and $f_{C_{2\varphi }}(c_{2\varphi })$ are shown follows, respectively:

(21) $$f_{\Delta \varphi }(\Delta \varphi )=\{\begin{array}{cc} \frac{1}{2\pi }+\frac{\Delta \varphi }{4\pi^{2}} & \left(-2\pi ,0\right)\\ \frac{1}{2\pi }-\frac{\Delta \varphi }{4\pi^{2}} & \left(0,2\pi \right) \end{array}$$

(22) $$f_{C_{2\varphi }}(c_{2\varphi })=\{\frac{1}{\pi \sqrt{\left(1+c_{2\varphi })(1-c_{2\varphi }\right)}}\;|c_{2\varphi }|\leq 1$$

According to (22), the expectation and variance of $C_{2\varphi }$ are provided:

(23) $$E\{C_{2\varphi }\}=\int_{-\infty }^{+\infty }\cos(2\Delta \varphi )f(\Delta \varphi )d\Delta \varphi =0$$

(24) $$VAR\{C_{2\varphi }\}=E\{C_{2\varphi }^{2}\}-E^{2}\{C_{2\varphi }\}=\int_{-\infty }^{+\infty }\cos^{2}(2\Delta \varphi )f(\Delta \varphi )d\Delta \varphi =0.5$$

From the above section, the distribution of FLD output in unlock situation is completely different from that in lock situation.

## 4. Comparison between Theoretical and Simulation Results of $C_{2\varphi }$

The statistics characteristic of $C_{2\varphi }$ has been discussed in the previous sections. It provides a threshold setting that indicates the lock/unlock condition of FLD. In order to validate the validity of the theoretical analysis, we will compare between theoretical and simulation results.

The probability density functions of $C_{2\varphi }$ under lock and unlock conditions can be obtained from (19) and (20), respectively. At the same time, Monte Carlo simulation experiments that software receiver [23] acquires and tracks simulated GPS L1 signal are carried out. The parameters of the simulation experiment are shown in Table 1.

The simulated GPS signal with 4.996 MHz intermediate frequency is generated by the software program. Four signals with different *C*/*N*_0_ values and a noise signal are simulated. The software receiver is used to process the signal and the probability density function (PDF) of FLD output can be retrieved under different situations.

**Figure 1:**
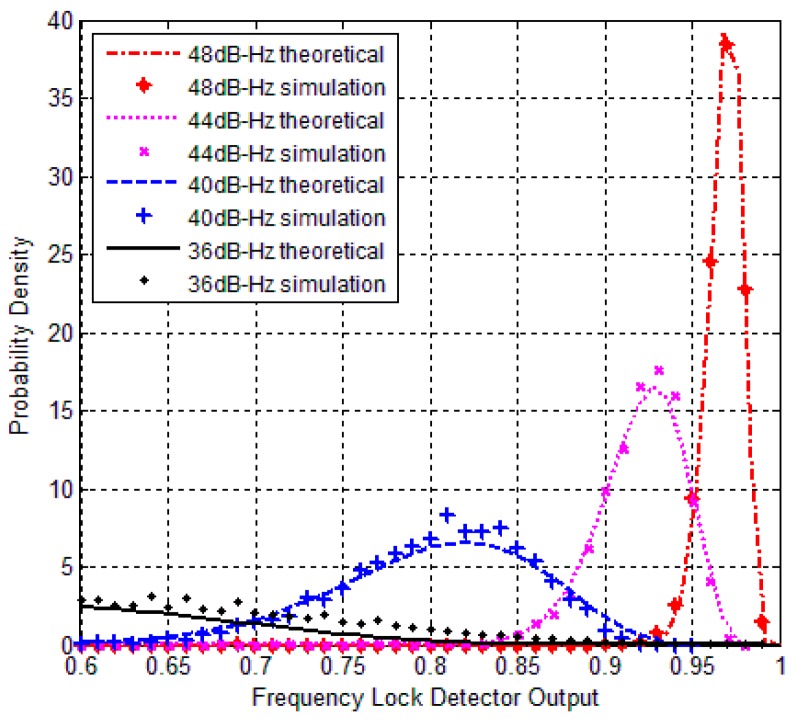
PDF of theoretical and simulation under high *C*/*N*_0_ (*T* = 1 ms, *M* = 20).

**Figure 2:**
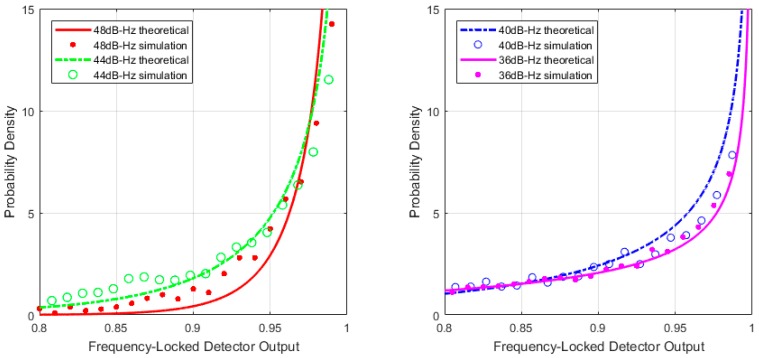
Probability density function (PDF) of theoretical and simulation under high *C*/*N*_0_ (*T* = 1 ms, *M* = 1).

**Figure 3:**
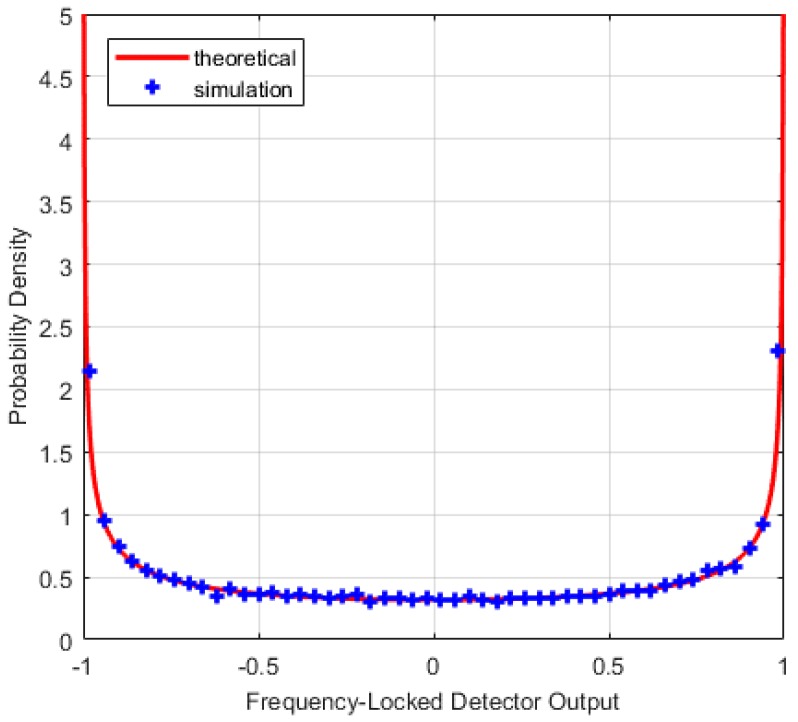
Frequency-locked detector (FLD) output probability density with no signal (*M* = 1).

The comparison between theoretical and simulation results are shown in Figure 1, Figure 2 and Figure 3. Figure 1 and Figure 2 show the PDF of $C_{2\varphi }$ with different average length *M* when signal presented. However, both of the figures indicate that maximum probability point of the horizontal axis moves closer to one when increasing the *C*/*N*_0_ values, which means that $\Delta \varphi_{i}$ become smaller. Normally, $C_{2\varphi }$ decreases when reducing *C*/*N*_0_. Maximum probability points in probability density curves change dramatically with different *C*/*N*_0_ values in Figure 1 but no evident differences in Figure 2. It means that probability density curve could not indicate $C_{2\varphi }$ changing with different *C*/*N*_0_ because of interference by noise when *M* = 1 in Figure 2. So, *M* should be set larger. Figure 3 shows $C_{2\varphi }$ obeys uniform distribution when no signal presented.

### 4.1. Lock Probability Analysis of FLD Output

**Figure 4:**
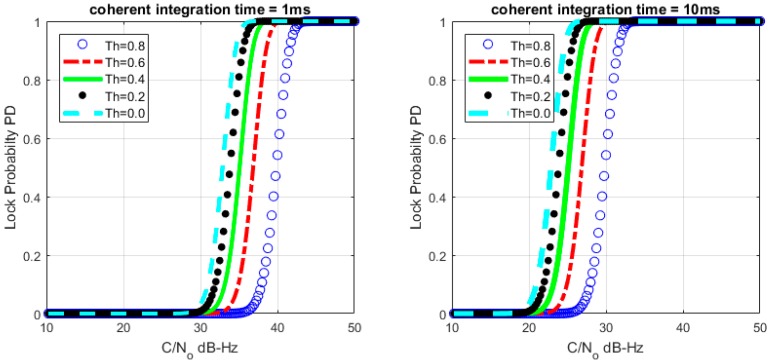
Loop lock probability under different *C*/*N*_0_ with *M* = 20.

**Figure 5:**
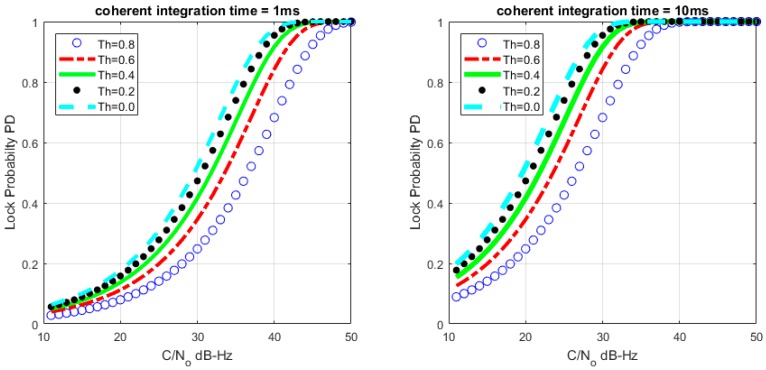
Loop lock probability under different *C*/*N*_0_ with *M* = 1.

**Figure 6:**
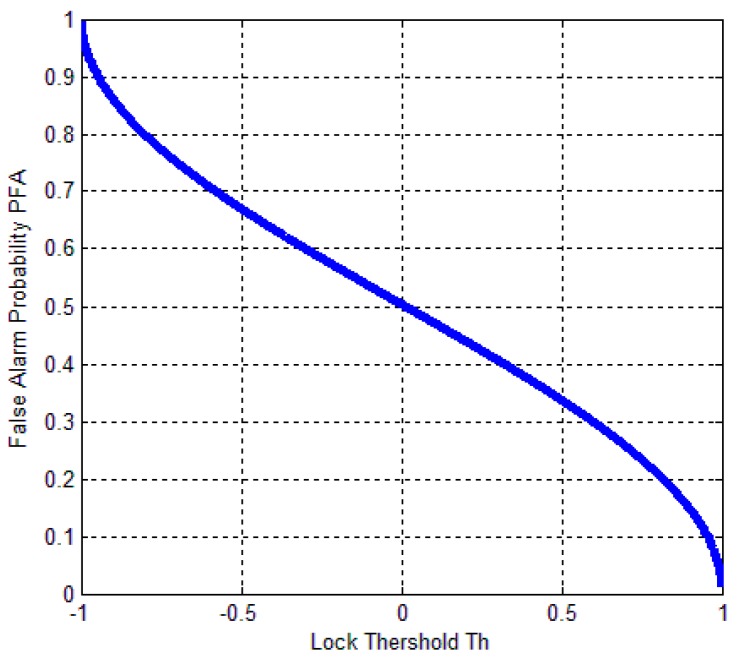
Relation between FLD false alarm probability and threshold (*Th*).

The output distribution of FLD is mainly related with the *C*/*N*_0_ of the received signal. With a given $C/N_{0}=\lambda$, the locked threshold of the tracking loop can be determined by the false alarm probability $P_{FA}$ and the lock probability $P_{D}$.

(25) $$P_{FA}=P\{C_{2\varphi }>Th|noise\;input\;only\;\}=\int_{Th}^{1}\frac{1}{\pi \sqrt{\left(1+c_{2\varphi })(1-c_{2\varphi }\right)}}dc_{2\varphi }$$

(26) $$\begin{array}{cc} P_{D} & =P\{C_{2\varphi }>Th|C/N_{0}=\lambda \}\\ & =P\{Y=\frac{M(1-C_{2\varphi })}{2\sigma^{2}}<\frac{M(1-Th)}{2\sigma^{2}}|C/N_{0}=\lambda \}\\ & =F_{Y}(\frac{M(1-Th)}{2\sigma^{2}}|C/N_{0}=\lambda ) \end{array}$$

where $F_{Y}(y)$ is the distribution function of the random variable Y($Y\sim \chi^{2}(M)$). Figure 4, Figure 5 and Figure 6 describe the relationships between the lock probability and the false alarm probability. As shown in Figure 4 and Figure 5, the lock probability increases with the increasing coherent integration time under the same *C*/*N*_0_. The lock probability can be also increased by decreasing the threshold, but the false alarm probability also increases under that situation. Figure 6 shows the relation between the FLD output false alarm probability and threshold. As a result, in order to set optimal FLD threshold, *C*/*N*_0_ and false alarm should be considered at same time.

### 4.2. Mean-Time-To-Lose-Lock (MTLL) of FLD

In order to evaluate the performance of the tracking loop, we need to analyze the mean-time-to-lose-lock (MTLL) of the frequency tracking loop. The time should be shorter when the signals are not present, while longer when the signals are present. The distinct threshold will influence how long the tracking loop can detect loop state changes. To be more specific, MTLL ($E\{T_{F}\}$) is used to indicate the average time required that tracking loop detect state changed from false alarm to correct detection under no signal condition. MTLL ($E\{T_{D}\}$) will imply that how long tracking loop change the signal detection state to missing detection state when the signal presented. Over a total time TT, MTLL ($E\{T_{F}\}$) under false alarm $P_{FA}$ can be obtained [14] as:

(27) $$\begin{array}{cc} E\{T_{F}\}{|}_{TT->\infty } & =\sum_{N=1}^{TT}NP_{FA}^{N}(1-P_{FA}){|}_{TT->\infty }\\ & =\frac{1}{1-P_{FA}} \end{array}$$

where the false alarm probability $P_{FA}$ with a certain threshold (*M* = 1) is shown in (25), *N* is time slot. MTLL ($E\{T_{F}\}$) indicates average time that frequency locked loop used to detect no signal state.

**Figure 7:**
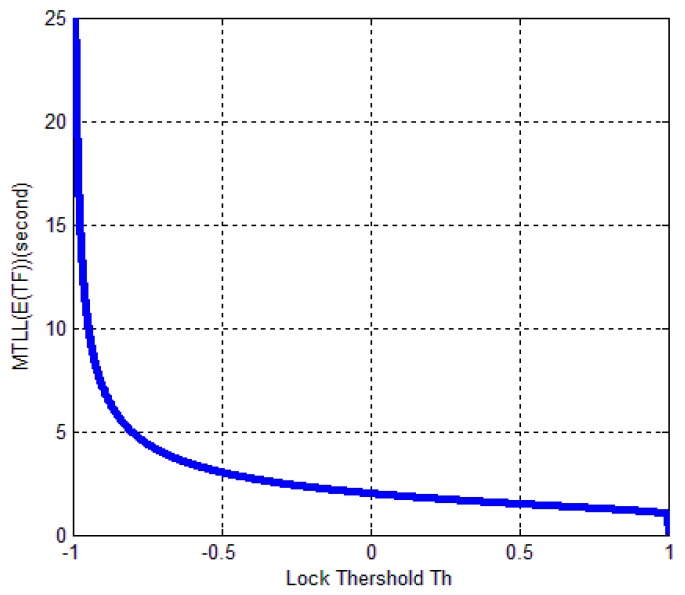
Relation between MTLL ($E\{T_{F}\}$) and threshold Th under no signal presented situation.

Based on (25) and (27), we can get the relationship between MTLL ($E\{T_{F}\}$) and lock threshold. As shown in Figure 7, it indicates that relation between MTLL ($E\{T_{F}\}$) and threshold under no signal situation. The continuous tracking time is gradually decreased when increasing Th. MTLL ($E\{T_{F}\}$) is almost same when Th above zero. The MTLL ($E\{T_{F}\}$) of false alarm in FLD shown in Figure 7 is similar with that in PLD in [14].

When the signals present, the MTLL ($E\{T_{D}\}$) can be obtained [14] as:

(28) $$E\{T_{D}\}{|}_{TT->\infty }=\frac{1}{1-P_{D}}$$

where the lock probability $P_{D}$ with a certain threshold Th is shown in (26). MTLL ($E\{T_{D}\}$) indicates the average time that the frequency locked loop takes to maintain signal detection state.

**Figure 8:**
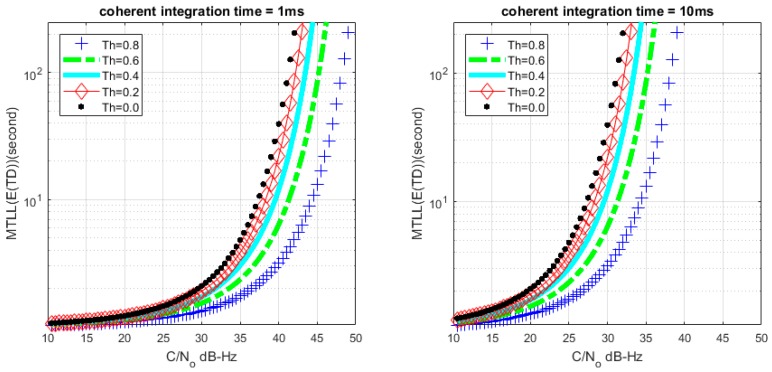
Relation between mean-time-to-lose-lock (MTLL) ($E\{T_{D}\}$) and *C*/*N*_0_ (*M* = 1) under signal presented situation.

**Figure 9:**
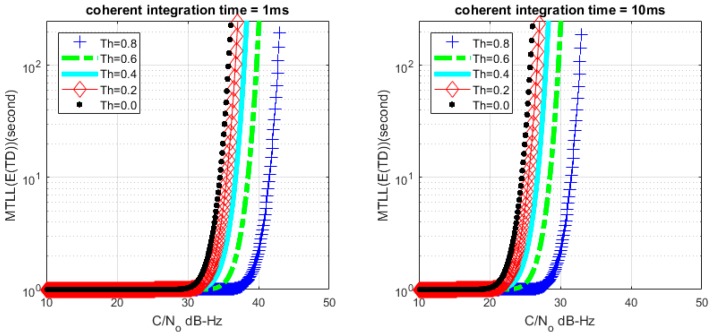
Relation between MTLL ($E\{T_{D}\}$) and *C*/*N*_0_ (*M* = 20) under signal presented situation.

**Figure 10:**
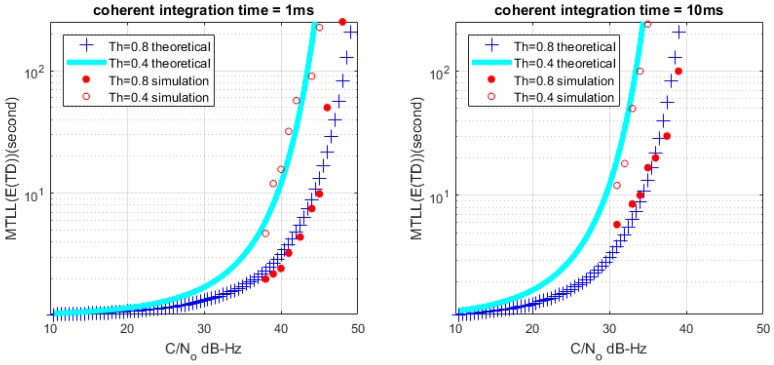
MTLL ($E\{T_{D}\}$) of theoretical and simulation *C*/*N*_0_ (*M* = 1).

**Figure 11:**
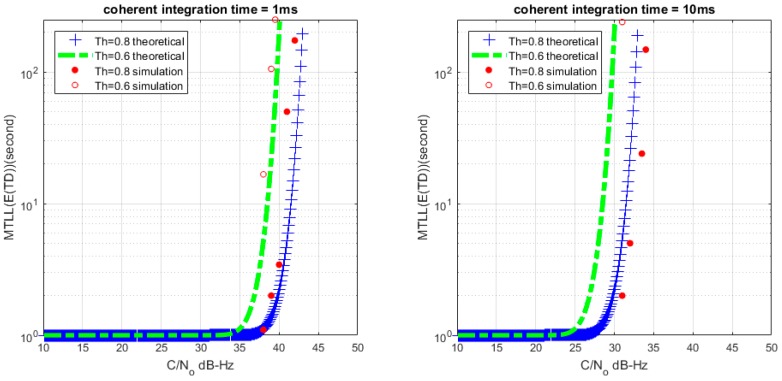
MTLL ($E\{T_{D}\}$) of theoretical and simulation *C*/*N*_0_ (*M* = 20).

Based on (26) and (28), we can get the relationship between MTLL ($E\{T_{D}\}$) and *C*/*N*_0_ with a certain lock threshold. The relationship curves are shown in Figure 8 and Figure 9 with *M* = 1 and *M* = 20, the continuous tracking time MTLL ($E\{T_{D}\}$) gradually increases when increasing *C*/*N*_0_ or coherent integration time. At the same time, the verification of MTLL is conducted with the help of Monte Carlo simulation in Figure 10 and Figure 11.

The theoretical results show that the MTLL ($E\{T_{F}\}$) is only 2 s when the signal does not present and the threshold Th is equal to 0 in Figure 7. Oversized noise can cause false alarm that leads to error lock in frequency locked loop. We can judge the error lock state in is 2 s. The MTLL ($E\{T_{D}\}$) is about 40 s when the integration time is 1 ms under signal with 40 dB-Hz *C*/*N*_0_, and 10 ms for the signal with 30 dB-Hz *C*/*N*_0_ that threshold Th is equal to 0 (*M* = 1) in Figure 8 when the signal present.

**Figure 12:**
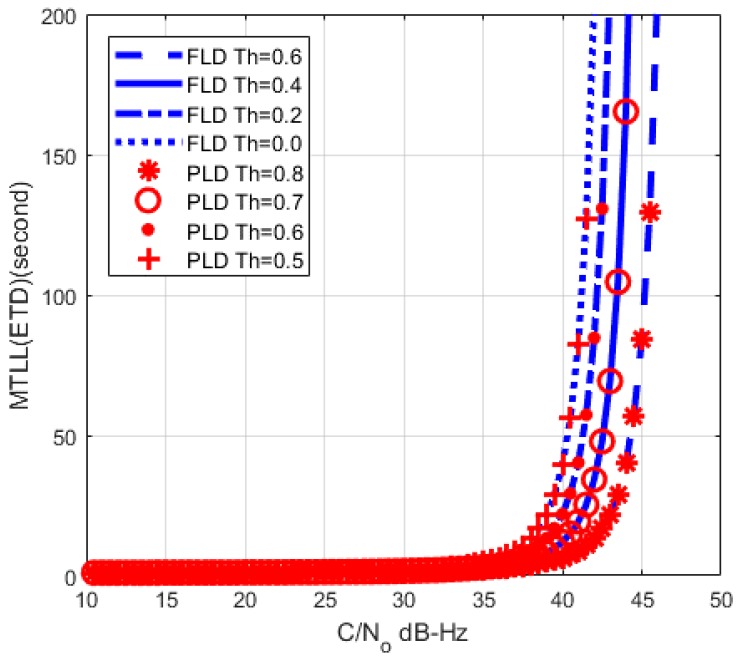
MTLL comparison between FLD (*M* = 1) and PLD.

The comparison with PLD [14] is shown in Figure 12. The threshold setting of PLD at 0.7 has a similar curve to the threshold setting of FLD at 0.4, while PLD at 0.6 is similar to the FLD at 0.2. It means that FLD has lower threshold than PLD under similar MTLL. Therefore, FLD can track signal more stable than PLD because of lower lock threshold. Also, the PLD threshold setting criteria experience cannot apply directly to FLD. These should be considered in the practical receiver implementation.

### 4.3. Setting Threshold with Actual Data

In order to set reasonable threshold of FLD, we can get the relationship curve about FLD output and *C*/*N*_0_ based on (6) and thermal jitter of FLL [24]:

(29) $$\sigma_{tFLL}=\frac{1}{2\pi T_{coh}}\sqrt{\frac{4FB_{L}}{C/N_{0}}(1+\frac{1}{T_{coh}*C/N_{0}})}$$

where $\sigma_{tFLL}$ is the frequency jitter caused by thermal noise, $B_{L}$ is equivalent noise bandwidth of tracking loop, F is equal to 1 and 2 for low, and high *C*/*N*_0_ individually. Normally, $3\sigma_{tFLL}$ is used to estimate tracking performance of FLL. The relationship between threshold of FLD and *C*/*N*_0_ based on (6) and (29) is

(30) $$Th_{C_{2\varphi }}=\frac{1}{M}\sum_{i=1}^{M}\cos(12\pi T_{coh}\sigma_{tFLL})$$

**Figure 13:**
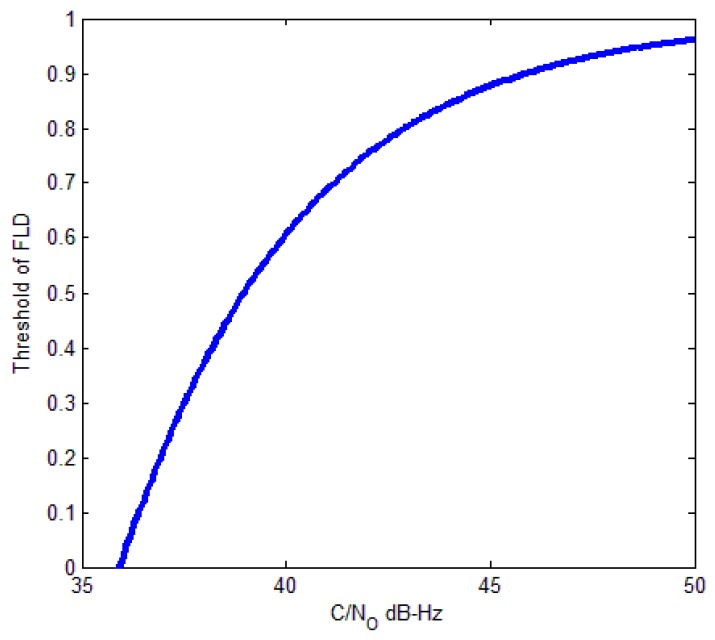
Relation between threshold of FLD and *C*/*N*_0_ (*M* = 20).

Assuming that *M* = 20, coherent integration length $T_{coh}=1\;\mathrm{ms}$, $B_{L}=25\;\mathrm{Hz}$, we can figure (30) in Figure 13.

**Figure 14:**
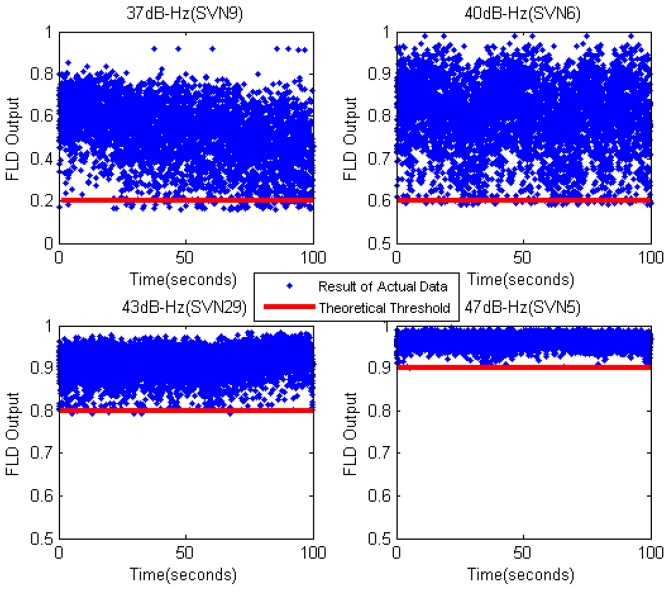
FLD output of actual data with different *C*/*N*_0_ (*M* = 20).

Then, we recorded actual real-time data samples from the intermediate frequency (IF) signal of GPS receiver. The data were recorded on building roof for a duration of 100 s at 62 million samples per second. The *C*/*N*_0_ of the GPS satellites were 37 dB-Hz (SVN 9), 40 dB-Hz (SVN 6), 43 dB-Hz (SVN 29), and 47 dB-Hz (SVN 5). From Figure 11, we can know that the theoretical threshold of FLD is 0.2 (37 dB-Hz), 0.6 (40 dB-Hz), 0.8 (43 dB-Hz), and 0.9 (47 dB-Hz). Figure 14 shows the comparison of FLD outputs and theoretical thresholds with different satellites. The results show that the theoretical thresholds fit well with FLD outputs of real signals, which provide a solid support for FLD threshold setting criteria.

## 5. Conclusions

The approximate probability distribution of frequency-locked detector output has been theoretically derived by statistical approach. The mean-time-to-lose-lock (MTLL) of the frequency tracking loop has been analyzed based on the statistical characteristic of the FLD output to evaluate the performance of the tracking loop, as well as to compare with the PLD output. MTLL is a kind of evaluation criteria to measure FLD or PLD performance. The relationship among mean-time-to-lose-lock, detection threshold, and *C*/*N*_0_ can be revealed with this method, which shows the difference between threshold settings in the phase-locked detector and the frequency-locked detector. The FLD threshold is, as demonstrated by the analysis results, much lower in comparison to the PLD threshold in the PLL with the same MTLL performance. The FLD output is an accumulation of adjacent periods, which means that FLD can be used to indicate a weaker signal to ensure the stability of the tracking loop. Therefore, a theoretical basis for the threshold setting in the frequency-locked detector of the GPS receiver is provided.

Meanwhile, the result of the experiment using the actual data indicates several suggested thresholds under different *C*/*N*_0_ values, which can be used as a reference for the threshold setting criteria of FLD in receiver.

## Figures and Tables

**Table 1:** Simulation parameters of software receiver.

Parameters	Value
Signal	GPS L1 Signal
Signal *C*/*N*_0_	High *C*/*N*_0_: 48 dB-Hz, 44 dB-Hz, 40 dB-Hz, 36 dB-Hz Low *C*/*N*_0_: No Signal
Signal sampling rate	12 MHz
Coherent integration length	1 ms
Doppler frequency	0 Hz
Simulation times	5000 times, 5 s (*M* = 1 *M* = 20)
